# The association between digital leadership and VUCA -related psychological and cognitive dimensions among healthcare professionals: a cross-sectional study

**DOI:** 10.1186/s12913-026-15023-x

**Published:** 2026-07-11

**Authors:** Gizem Şahin, Nurcan Coşkun Us

**Affiliations:** 1 Department of Health Management, Ondokuz Mayıs University, Samsun, Türkiye; 2 Health Management Department, Faculty of Health Sciences, Ondokuz Mayıs University, Samsun, Türkiye

**Keywords:** Digital leadership, VUCA, Healthcare professionals, Resistance to change, Intolerance of uncertainty, Cognitive flexibility, Ambiguity tolerance

## Abstract

**Background:**

Healthcare organizations increasingly operate in volatile, uncertain, complex, and ambiguous (VUCA) environments, requiring leadership approaches that facilitate adaptation and resilience. Although digital leadership has gained growing attention, evidence regarding its association with VUCA-related psychological and cognitive dimensions, such as resistance to change, intolerance of uncertainty, cognitive flexibility, and ambiguity tolerance, remains limited among healthcare professionals.

**Aim:**

This study examined the association between digital leadership and perceived VUCA-related psychological and cognitive dimensions among healthcare professionals.

**Method:**

A cross-sectional study was conducted among 495 healthcare professionals working in three hospitals in Muğla, Türkiye. Data were collected using validated instruments measuring digital leadership, resistance to change, intolerance of uncertainty, cognitive flexibility, and ambiguity tolerance. Descriptive statistics, Pearson correlation, and linear regression analyses were performed.

**Results:**

Digital leadership was negatively associated with resistance to change (*r* = − 0.550, *p* < 0.001) and intolerance of uncertainty (*r* = − 0.547, *p* < 0.001). Positive associations were observed between digital leadership and cognitive flexibility (*r* = 0.466, *p* < 0.001) as well as ambiguity tolerance (*r* = 0.500, *p* < 0.001). Regression analyses showed that digital leadership was significantly associated with all examined VUCA-related psychological and cognitive dimensions.

**Conclusion:**

Higher perceptions of digital leadership were associated with lower resistance to change and uncertainty intolerance, and with higher cognitive flexibility and ambiguity tolerance among healthcare professionals. These findings suggest that digital leadership may support adaptive responses in healthcare environments characterized by uncertainty and complexity.

## Introduction

Digital leadership refers to an integrated set of competencies that combine digital market knowledge with strategic leadership skills such as decision-making, communication, and coordination to enable effective performance in digital ecosystems [1]. In healthcare organizations, accelerating digital transformation has intensified the need for adaptive leadership approaches that ensure continuity, efficiency, and flexibility in service delivery [2].

As healthcare organizations continue to adopt digital technologies and data-driven systems, they increasingly operate in environments characterized by rapid change and uncertainty. These conditions are commonly described through the VUCA framework, which encompasses volatility, uncertainty, complexity, and ambiguity [3, 4]. Specifically, volatility reflects rapid and unexpected changes, uncertainty refers to limited predictability of future events, complexity involves multiple interacting variables within systems, and ambiguity represents unclear cause–effect relationships [5, 6].

Importantly, VUCA conditions are not only organizational characteristics but also factors that influence how employees perceive, interpret, and respond to their work environment. In healthcare settings, exposure to uncertainty and complexity may affect decision confidence, adaptive capacity, and the ability to process information effectively. Consequently, understanding the factors that may shape employees’ responses to VUCA conditions has become increasingly important.

Among these factors, leadership has been identified as a critical mechanism through which organizations support employee adaptation in uncertain environments. Digital leadership, in particular, may play an important role by strengthening employees’ ability to process information, adapt to change, and engage in data-driven decision-making. Leaders who effectively integrate digital tools into organizational practices may reduce perceived uncertainty, improve sense-making processes, and support greater cognitive flexibility when employees are confronted with complex and rapidly changing situations. Therefore, digital leadership can be theoretically linked to how VUCA conditions are perceived and cognitively managed by healthcare professionals.

This relationship may be particularly relevant in healthcare organizations, where digital transformation occurs alongside high levels of clinical uncertainty and organizational complexity. Healthcare professionals routinely operate within multidisciplinary care systems, rapidly evolving technological environments, and unpredictable patient-care processes. In such settings, volatility may emerge through changing patient demands and technological innovations, uncertainty through unpredictable clinical outcomes, complexity through interconnected healthcare systems, and ambiguity through differing interpretations of digital health solutions [7]. Taken together, these characteristics make healthcare an especially appropriate context for examining the association between digital leadership and healthcare professionals’ psychological and cognitive responses to VUCA-related conditions.

In the present study, VUCA conditions were not measured directly as four independent dimensions. Instead, resistance to change, intolerance of uncertainty, cognitive flexibility, and ambiguity tolerance were used as proxy indicators reflecting healthcare professionals’ psychological and cognitive responses to VUCA-related work environments. Accordingly, the term “VUCA-related dimensions” is used throughout the manuscript to refer to these response-oriented constructs rather than the VUCA framework itself.

## Theoretical framework and hypothesis development

### Digital leadership and VUCA conditions in healthcare

Digital leadership can be conceptualized as an organizational capability that enables leaders to guide employees, teams, and institutions through digital transformation processes by combining strategic vision, technological understanding, data-informed decision-making, and effective communication. In healthcare organizations, the relevance of digital leadership has increased due to the rapid diffusion of digital health technologies, electronic health records, telemedicine, data-driven decision systems, and digitally mediated workflows. Leadership in digital health services is therefore not limited to the technical implementation of technologies; rather, it also involves creating the conditions that enable employees to adapt to new work processes, interpret digital information, and maintain service continuity in complex environments.

Healthcare systems increasingly operate under volatile, uncertain, complex, and ambiguous conditions. VUCA conditions are especially relevant to healthcare because clinical workflows, patient needs, technological change, interprofessional coordination, and organizational decision-making are often characterized by unpredictability and complexity. Recent healthcare literature emphasizes that responding to VUCA conditions requires vision, understanding, clarity, and agility, which are closely aligned with the core functions of leadership in digitally transforming organizations. Accordingly, digital leadership may represent an important contextual factor associated with healthcare professionals’ psychological and cognitive responses to uncertainty, complexity, and ambiguity in their work environment.

### Review of previous studies and research gap

Previous studies have examined digital leadership in relation to organizational performance, innovation capability, digital culture, employee digital capabilities, organizational agility, and digital intensity. For example, Benitez et al. [1] demonstrated that digital leadership capability contributes to innovation performance through platform digitization in European firms. Shin et al. [8] found that digital leadership was associated with digital culture and employees’ digital capabilities in South Korean organizations. In healthcare, Laukka et al. [9] emphasized the importance of leadership in digital health services, while Kludacz-Alessandri et al. [10] showed that digital transformational leadership was associated with digital intensity among primary healthcare entities through organizational agility.

Although these studies provide important evidence regarding the organizational relevance of digital leadership, fewer studies have examined how digital leadership is associated with employees’ psychological and cognitive responses to VUCA-related conditions. This gap is particularly important in healthcare settings, where professionals are required to adapt to rapid digitalization while simultaneously managing uncertainty, ambiguity, and complex patient-care processes. Therefore, the present study extends existing digital leadership and VUCA literature by examining the associations between digital leadership and VUCA-related psychological and cognitive dimensions, namely resistance to change, intolerance of uncertainty, ambiguity tolerance, and cognitive flexibility, among healthcare professionals.

### Hypothesis development

Digital leadership, characterized by vision-driven digital transformation, data-informed decision-making, and effective communication in technology-mediated environments, is expected to be negatively associated with employees’ resistance to change. Leadership behaviors that provide clarity, support, and strategic direction may reduce psychological barriers to organizational change processes. Empirical research in digital transformation contexts supports this assumption. Weber et al. [11], in an experimental study, showed that complementary leadership behaviors are important in guiding employees through digital transformation and shaping their responses to organizational change. Similarly, Shin et al. [8] demonstrated that digital leadership is associated with digital culture and employee digital capabilities, both of which are relevant for employee adaptation to technology-driven change. In the healthcare context, Laukka et al. [9] and Kludacz-Alessandri et al. [10] further highlight the importance of leadership in supporting digital transformation and organizational agility. Accordingly, the following hypothesis is proposed:

H1: Digital leadership is negatively associated with resistance to change.

Intolerance of uncertainty reflects individuals’ difficulty in coping with unpredictable, unclear, or insufficiently structured situations. In digitally transforming healthcare organizations, uncertainty may arise from changing technologies, new workflows, unclear role expectations, and unpredictable clinical or organizational demands. Previous research on VUCA environments suggests that uncertainty and unpredictability require leadership practices that provide clarity, information flow, and adaptive direction. Nowacka and Rzemieniak, [4] showed that VUCA conditions influence the digital competence requirements of managers in the power industry, indicating the importance of leadership and competence development under uncertainty. In healthcare, Cernega et al. [7] emphasized that responding to VUCA conditions requires vision, understanding, clarity, and agility. These findings suggest that digital leadership may be associated with lower intolerance of uncertainty by helping employees interpret and manage uncertain work conditions more effectively. Therefore, the following hypothesis is proposed:

H2: Digital leadership is negatively associated with uncertainty intolerance.

Ambiguity tolerance refers to the capacity to function effectively when information is incomplete, unclear, or open to multiple interpretations. Healthcare professionals frequently encounter ambiguous situations due to evolving digital tools, complex patient-care pathways, and uncertain organizational priorities. Studies on VUCA and digital transformation indicate that employees’ ability to tolerate ambiguity is important for adaptation in complex environments. Horstmeyer, [6] emphasized that curiosity and soft skills may help employees navigate ambiguity and rapidly changing work conditions. Cernega et al. [7] similarly argued that clarity and agility are essential for addressing ambiguity in healthcare environments. Digital leadership may contribute to ambiguity tolerance by improving communication, supporting sense-making, and encouraging data-informed decision-making. Accordingly, the following hypothesis is proposed:

H3: Digital leadership is positively associated with ambiguity tolerance.

Cognitive flexibility refers to individuals’ ability to shift perspectives, generate alternative interpretations, and adapt their thinking and behavior in response to changing environmental demands. Digital leadership may foster cognitive flexibility by encouraging learning, innovation, experimentation, and adaptive problem-solving. Empirical evidence from different contexts supports the association between digital leadership and adaptive employee outcomes. Benitez et al. [1] found that digital leadership capability supports innovation performance through platform digitization. Gao et al. [12] reported that digital leadership is associated with employee innovative behavior through psychological empowerment. Shin et al. [8] showed that digital leadership is related to employees’ digital capabilities, while Kludacz-Alessandri et al. [10] demonstrated that digital transformational leadership is associated with organizational agility in primary healthcare entities. These findings suggest that digital leadership may be positively associated with cognitive flexibility among healthcare professionals. Thus, the following hypothesis is proposed:

H4: Digital leadership is positively associated with cognitive flexibility.

Finally, cognitive flexibility is considered a key psychological resource that facilitates adaptive responses to VUCA-related conditions. Individuals with higher cognitive flexibility are more capable of reinterpreting complex situations, shifting between alternative perspectives, and adapting their responses when faced with incomplete or changing information. VUCA-related studies suggest that adaptive learning, curiosity, and flexible thinking are important for navigating ambiguity and uncertainty in contemporary work environments. Guo et al. [13], in a study of tourism and hospitality students in China, found that perceived outcome-based education was associated with VUCA skills, highlighting the importance of adaptive learning capacities in developing responses to uncertainty and complexity. Horstmeyer, [6] also emphasized the role of curiosity and soft skills in navigating ambiguous work contexts. Therefore, healthcare professionals with higher cognitive flexibility may be more likely to tolerate ambiguous situations effectively. Accordingly, the following hypothesis is proposed:

H5: Cognitive flexibility is positively associated with ambiguity tolerance.

### Research model

**Fig. 1:**
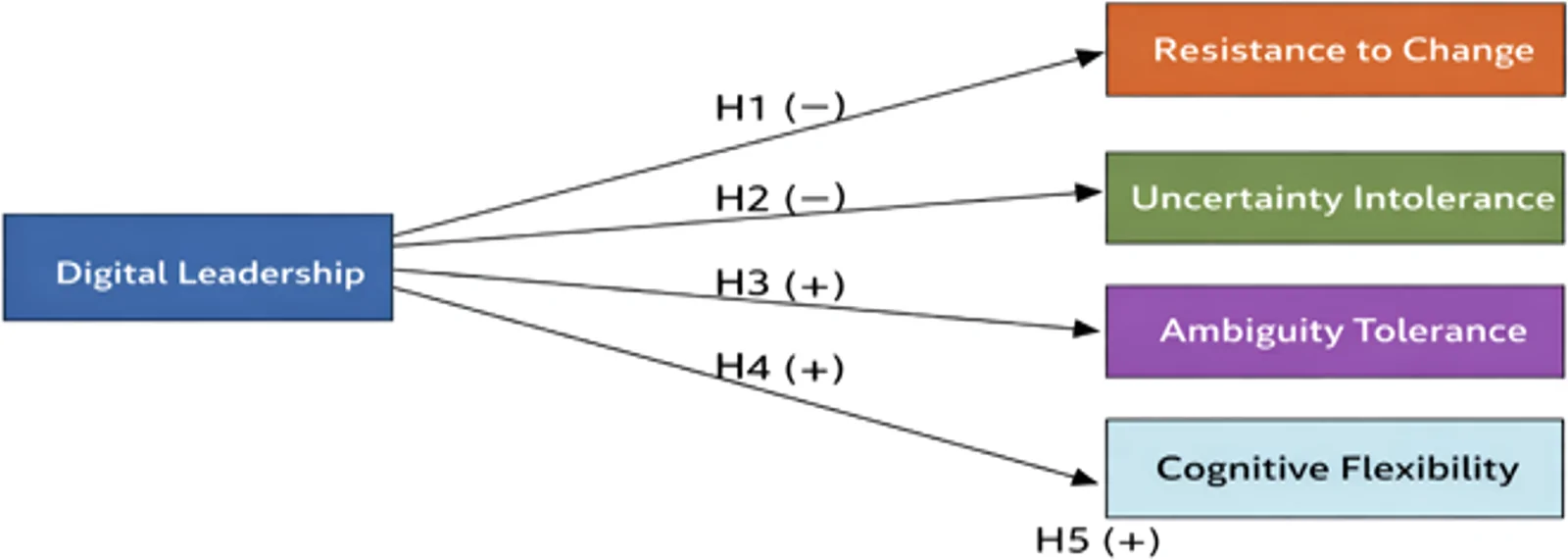
Research model

Following the theoretical framework, review of previous studies, research gap, and hypothesis development, the research model is presented in Fig. 1. In this model, digital leadership is positioned as the primary independent variable. The model proposes that digital leadership is negatively associated with resistance to change and uncertainty intolerance, and positively associated with ambiguity tolerance and cognitive flexibility. In addition, cognitive flexibility is proposed to be positively associated with ambiguity tolerance. Overall, the model provides an integrated representation of the hypothesized relationships and highlights the potential role of digital leadership in shaping healthcare professionals’ psychological and cognitive responses to VUCA-related conditions.

**Fig. 2:**
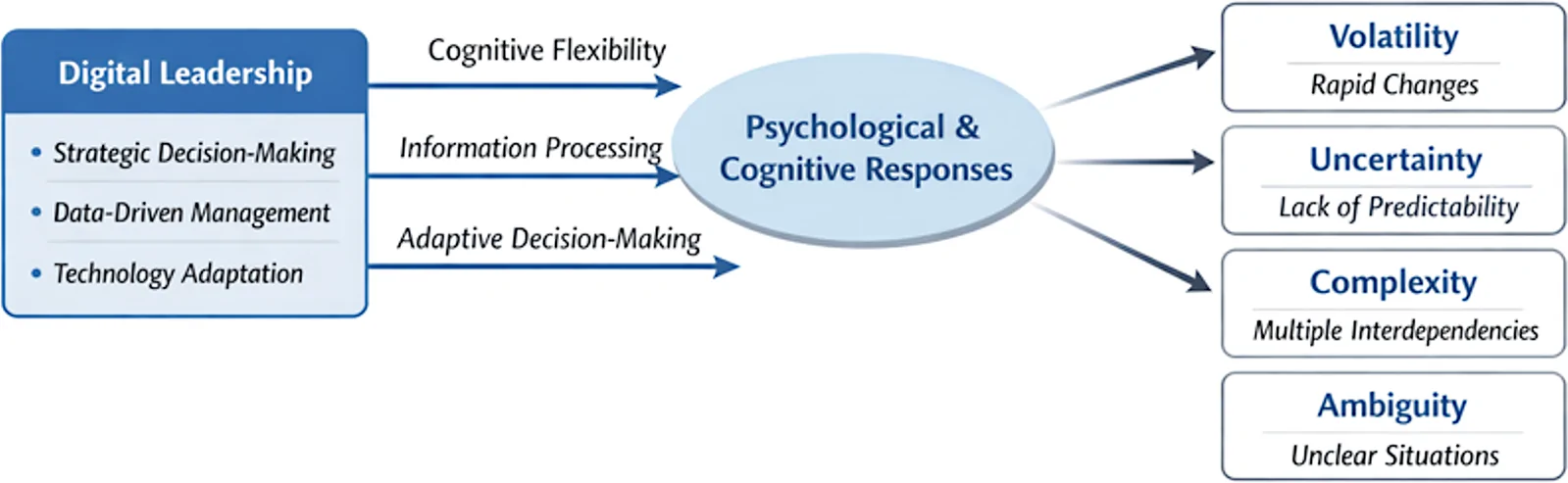
Conceptual framework of digital leadership and VUCA-related psychological and cognitive responses

Figure 2 presents the conceptual relationships between digital leadership and VUCA-related psychological and cognitive responses. In the model, digital leadership is treated as the primary independent variable, and its associations with healthcare professionals’ responses to VUCA-related conditions are examined.

## Materials and methods

### Type of research

This study employed a quantitative, cross-sectional research design to examine the relationships between digital leadership and VUCA-related psychological and cognitive dimensions among healthcare professionals. The general survey model was used, which is commonly applied in social science research to collect data on individuals’ attitudes, perceptions, and behaviors at a specific point in time [14].

### Population and sample

The study was conducted among healthcare professionals working in three hospitals located in the Marmaris district of Muğla, Türkiye. Data were collected through face-to-face questionnaires between February 1 and May 1, 2024. The required sample size was calculated as 474 using Cochran’s formula (Cochran, 1977). A total of 600 questionnaires were initially collected; however, after excluding incomplete responses and questionnaires lacking informed consent, 495 valid questionnaires were retained for analysis. Participants were recruited using a convenience sampling method (Saunders et al., 2016), a non-probability sampling technique that facilitated data collection under field conditions. Inclusion criteria were active employment in one of the participating hospitals and voluntary consent to participate. Although convenience sampling enabled efficient access to healthcare professionals, it may limit sample representativeness and introduce potential sampling bias; therefore, caution should be exercised when generalizing the findings beyond the study population.

### Data collection tools

Data were collected using a structured questionnaire consisting of two main sections: demographic information and validated measurement scales.

The demographic section included variables such as gender, age, occupation, education level, income level, work unit, tenure in the profession and institution, marital status, and prior awareness of the concept of VUCA.

### Measurement scales

The study employed previously validated scales to ensure measurement reliability and validity.

### Digital leadership scale

Digital leadership was measured using the scale developed by Claassen et al. [15] and adapted into Turkish by Bilginoğlu and Yozgat [16]. The scale consists of items measured on a 6-point Likert-type format. In the present study, the Cronbach’s alpha coefficient was 0.95, indicating excellent internal consistency.

### VUCA-related psychological and cognitive dimensions

VUCA-related psychological and cognitive dimensions were operationalized using four validated scales combined in Koç et al. [17]: resistance to change [18], uncertainty intolerance [19], cognitive flexibility [20], and ambiguity tolerance Type-II [21].

Resistance to Change: 11 items, 7-point Likert scale; Cronbach’s alpha = 0.98

Uncertainty Intolerance: two sub-dimensions (prospective anxiety and inhibitory anxiety), 5-point Likert scale; Cronbach’s alpha = 0.95

Cognitive Flexibility: 8 items, 6-point Likert scale; Cronbach’s alpha = 0.97

Ambiguity Tolerance: 8 items, 5-point Likert scale; Cronbach’s alpha = 0.82

All scales demonstrated high reliability in the present study.

### Statistical analysis

Data were analyzed using IBM SPSS Statistics 22.0. Prior to analysis, assumptions of normality were evaluated using Kolmogorov–Smirnov test as well as skewness and kurtosis values.

Independent samples t-test and one-way ANOVA were used for group comparisons, followed by Bonferroni post-hoc tests where appropriate. Pearson correlation analysis was conducted to examine relationships among variables. The associations between digital leadership and the examined VUCA-related psychological and cognitive dimensions were evaluated using simple linear regression analyses. These models were unadjusted and examined bivariate associations between digital leadership and each outcome variable. Demographic and professional characteristics, including age, occupation, work unit, professional experience, and prior awareness of VUCA, were not included as covariates. Therefore, the findings should be interpreted as unadjusted associations rather than independent effects of digital leadership. Statistical significance was set at *p* < 0.05.

## Results

According to the descriptive statistics, the majority of the participants were female (64.44%), indicating a female-dominated sample structure. The age distribution was relatively balanced across groups, with the highest proportion of participants aged 40–49 (29.09%). In terms of occupational distribution, administrative unit staff constituted the largest group (32.12%), followed by nurses/midwives (26.46%), suggesting a heterogeneous professional composition.

Regarding professional experience, a dual concentration was observed: 34.34% of participants had 22 years or more of experience, while 42.42% had 0–4 years of experience, indicating a polarized structure between early-career and highly experienced professionals. Income distribution showed that the majority of participants (40.61%) earned between 25,001 and 40,000 TL, reflecting a mid-income clustering within the sample.

In terms of hospital units, a clear dominance of medical units was observed (75.56%), which may indicate that the findings are more strongly shaped by clinical service environments. Finally, the vast majority of participants (88.28%) reported that they had not heard of the concept of “VUCA” before, suggesting a generally low level of prior awareness regarding the concept (Table 1).

**Table 1:** Descriptive characteristics of the participants

Variable	Group	*n*	%
Gender	Female	319	64.44
	Male	176	35.56
Age	20–29	140	28.28
	30–39	121	24.44
	40–49	144	29.09
	50 and above	90	18.18
Occupation	Physician	82	16.57
	Nurse - Midwife	131	26.46
	Administrative Unit Staff	159	32.12
	Health Technician	123	24.85
Years of professional experience	0–7 years	121	24.44
	8–14 years	120	24.24
	15–21 years	84	16.97
	22 years and above	170	34.34
The participant’s seniority at a previous workplace	0–4 years	210	42.42
	5–9 years	95	19.19
	10–14 years	92	18.59
	15 years and above	98	19.80
Income Status	17.000–25.000 TL	151	30.51
	25.001-40.000 TL	201	40.61
	40.001 TL and above	143	28.89
Hospital Work Units	Medical Units	374	75.56
	Diagnostic Units	46	9.29
	Administrative Services Units	75	15.15
VUCA awareness	Yes	58	11.72
	No	437	88.28

TL=Turkish Liras

As a result of the correlation analyses conducted to examine the relationships between the scales and sub-dimensions, moderate and statistically significant negative relationships were found between the Resistance to Change scale and the Type II Multi-Stimulus Ambiguity Tolerance scale (*r* = − 0.582, *p* < 0.001), the Cognitive Flexibility scale (*r* = − 0.527, *p* < 0.001) and the Digital Leadership scale (*r* = − 0.550, *p* < 0.001) (Table 2). Similarly, significant negative relationships were found between Inhibitory Anxiety and Digital Leadership (*r* = − 0.537, *p* < 0.001) and between Prospective Anxiety and Digital Leadership (*r* = − 0.517, *p* < 0.001). On the other hand, moderate positive correlations were found between Type II Multiple Stimulus Ambiguity Tolerance (*r* = 0.500, *p* < 0.001) and Cognitive Flexibility (*r* = 0.466, *p* < 0.001) scores and Digital Leadership scores (Table 2). This suggests that digital leadership behaviors tend to increase as ambiguity tolerance and cognitive flexibility levels increase.

**Table 2:** Findings regarding the relationship between scale and sub-dimension scores

		Scale of resistance to change	Disabling anxiety	Forward-looking anxiety	Uncertainty intolerance scale general	Type II multi-stimulus ambiguity tolerance scale	Cognitive flexibility scale	Digital leadership scale
Scale of resistance to change	r	1.00	0.564^**^	0.520^**^	0.566^**^	− 0.582^**^	− 0.527^**^	− 0.550^**^
	p		< 0.001	< 0.001	< 0.001	< 0.001	< 0.001	< 0.001
Disabling anxiety	r	0.564^**^	1.00	0.865^**^	0.982^**^	− 0.611^**^	− 0.459^**^	− 0.537^**^
	p	< 0.001		< 0.001	< 0.001	< 0.001	< 0.001	< 0.001
Forward-looking anxiety	r	0.520^**^	0.865^**^	1.00	0.944^**^	− 0.580^**^	− 0.417^**^	− 0.517^**^
	p	< 0.001	< 0.001		< 0.001	< 0.001	< 0.001	< 0.001
Uncertainty intolerance scale general	r	0.566^**^	0.982^**^	0.944^**^	1.00	− 0.619^**^	− 0.458^**^	− 0.547^**^
	p	< 0.001	< 0.001	< 0.001		< 0.001	< 0.001	< 0.001
Type II multi-stimulus ambiguity tolerance scale	r	− 0.582^**^	− 0.611^**^	− 0.580^**^	− 0.619^**^	1.00	0.567^**^	0.500^**^
	p	< 0.001	< 0.001	< 0.001	< 0.001		< 0.001	< 0.001
Cognitive flexibility scale	r	− 0.527^**^	− 0.459^**^	− 0.417^**^	− 0.458^**^	0.567^**^	1.00	0.466^**^
	p	< 0.001	< 0.001	< 0.001	< 0.001	< 0.001		< 0.001
Digital leadership scale	r	− 0.550^**^	− 0.537^**^	− 0.517^**^	− 0.547^**^	0.500^**^	0.466^**^	1.00
	p	< 0.001	< 0.001	< 0.001	< 0.001	< 0.001	< 0.001	

Correlation coefficients were interpreted according to Gürbüz and Şahin [22]: |r| ≥ 0.70 = strong, 0.30–0.69 = moderate, and < 0.30 = weak association

In line with the significant relationships obtained from the correlation analysis, simple linear regression analyses were performed to examine the associations between digital leadership and the relevant outcome variables. Below are the results of the models established for each dependent variable. The regression model examining the association between digital leadership and resistance to change was found to be statistically significant [F(1, 493) = 214.32, *p* < 0.001]. Digital leadership scores were significantly associated with resistance to change scores in the unadjusted regression model (*p* < 0.001). Every 1-unit increase in digital leadership scores is associated with a 0.91-unit decrease in the resistance to change score. The model accounted for 30.2% of the variance in resistance to change (Table 3).

**Table 3:** Unadjusted linear regression analysis of the association between digital leadership and resistance to change

Dependent Variable: Resistance to Change Scores					
	B	Std. Error	Beta	t	*p*
Constant	65.28	2.01		32.44	< 0.001
Digital Leadership Scale	-0.91	0.06	-0.55	-14.64	< 0.001

F(1, 493) = 214.32, *p* < 0.001, *R* = 0.55, R² = 0.303, Adjusted R² = 0.302, Durbin–Watson = 1.73

The regression model examining the association between digital leadership and uncertainty intolerance was found to be statistically significant [F (1, 493) = 210.32, *p* < 0.001]. Digital leadership scores were significantly associated with uncertainty intolerance scores in the unadjusted regression model (*p* < 0.001). A 1-unit increase in digital leadership scores was associated with a 0.64-unit decrease in uncertainty intolerance scores. The model accounted for 29.8% of the variance in uncertainty intolerance scores (Table 4).

**Table 4:** Unadjusted linear regression analysis of the association between digital leadership and intolerance of uncertainty

Dependent Variable: Uncertainty Intolerance Scale General					
	B	Std. Error	Beta	t	*p*
Constant	52.65	1.42		36.96	< 0.001
Digital Leadership Scale	-0.64	0.04	-0.55	-14.50	< 0.001

F(1, 493) = 210.32, *p* < 0.001, *R* = 0.547, R² = 0.299, Adjusted R² = 0.298, Durbin–Watson = 1.73

The regression model examining the association between digital leadership and Type II Multi-Stimulus Ambiguity Tolerance was found to be statistically significant [F(1, 493) = 164.37, *p* < 0.001]. Digital leadership scores were significantly associated with Type II Multi-Stimulus Ambiguity Tolerance scores in the unadjusted regression model (*p* < 0.001). A 1-unit increase in digital leadership scores was associated with a 0.34-unit increase in Type II Multi-Stimulus Ambiguity Tolerance scores. The model accounted for 24.9% of the variance in Type II Multi-Stimulus Ambiguity Tolerance scores (Table 5).

**Table 5:** Unadjusted linear regression analysis of the association between digital leadership and type II multi-stimulus ambiguity tolerance

Dependent Variable: Type II Multi-Stimulus Ambiguity Tolerance Scale					
	B	Std. Error	Beta	t	*p*
Constant	15.64	0.85		18.36	< 0.001
Digital Leadership Scale	0.34	0.03	0.50	12.82	< 0.001

F(1, 493) = 164.37, *p* < 0.001, *R* = 0.50, R² = 0.250, Adjusted R² = 0.249, Durbin–Watson = 1.93

The regression model examining the association between digital leadership and cognitive flexibility was found to be statistically significant [F(1, 493) = 135.50, *p* < 0.001]. Digital leadership scores were significantly associated with cognitive flexibility scores in the unadjusted regression model (*p* < 0.001). A 1-unit increase in digital leadership scores was associated with a 0.32-unit increase in cognitive flexibility scores. The model accounted for 21.5% of the variance in cognitive flexibility scores (Table 6).

**Table 6:** Unadjusted linear regression analysis of the association between digital leadership and cognitive flexibility

Dependent Variable: Cognitive Flexibility Scale					
	B	Std. Error	Beta	t	*p*
Constant	29.65	0.90		32.91	< 0.001
Digital Leadership Scale	0.32	0.03	0.47	11.68	< 0.001

F(1, 493) = 135.50, *p* < 0.001, *R* = 0.466, R² = 0.217, Adjusted R² = 0.215, Durbin–Watson = 1.76

## Discussion

This study examined the association between digital leadership and healthcare professionals’ responses to VUCA-related psychological and cognitive dimensions, including resistance to change, uncertainty intolerance, ambiguity tolerance, and cognitive flexibility. The findings suggest that digital leadership is associated with adaptive behavioral and cognitive tendencies in healthcare environments characterized by complexity and continuous change.

The observed relationships are generally consistent with prior studies emphasizing the role of digital leadership in facilitating organizational adaptation during digital transformation processes [9, 23, 24]. However, the present study extends this literature by situating these relationships within healthcare settings, where organizational pressures such as high workload, resource constraints, and post-pandemic restructuring may intensify the need for adaptive leadership mechanisms.

One key finding is the association between digital leadership and reduced resistance to change. This result aligns with leadership literature suggesting that effective leadership can reduce behavioral resistance during organizational transformation processes [8, 25, 26]. In contrast, some studies in high-demand healthcare environments have reported persistent resistance despite leadership interventions, largely attributed to workload intensity and staffing shortages. This suggests that organizational conditions may moderate the effectiveness of leadership behaviors.

Similarly, the associations between digital leadership and cognitive flexibility and ambiguity tolerance indicate that individuals with higher adaptive cognitive resources tend to report more favorable perceptions of digital leadership. While this finding supports previous research highlighting the role of cognitive resources in digital transformation contexts [10, 27], it also raises the possibility that organizational culture may shape both leadership perceptions and cognitive adaptation simultaneously. In cultures characterized by openness to innovation and learning, such relationships may be strengthened.

Ambiguity tolerance appears to be particularly relevant in explaining adaptation to digital leadership. Rather than eliminating ambiguity, digital leadership may contribute to structuring information flow and decision-making processes, thereby reducing perceived uncertainty. This interpretation is consistent with studies emphasizing the importance of communication clarity and trust in leadership during uncertain organizational conditions [4, 6]. However, in contexts where communication overload or fragmented digital systems exist, ambiguity may persist despite leadership efforts.

The relationship between cognitive flexibility and digital leadership further suggests that adaptive cognitive capacity is closely linked with openness to digital leadership practices. Previous studies have reported similar findings in healthcare and educational contexts [13, 28]. Nevertheless, contrasting evidence indicates that high workload and time pressure may limit cognitive flexibility regardless of leadership style, suggesting that structural working conditions may override individual-level cognitive resources in some cases.

From a broader perspective, the regression results suggest that digital leadership is more strongly associated with behavioral responses such as resistance to change than with deeper cognitive constructs. This pattern may indicate that leadership influence is initially expressed through behavioral adaptation, while cognitive restructuring may require longer-term exposure and supportive organizational conditions.

Importantly, uncertainty intolerance findings highlight the relevance of leadership in shaping employees’ ability to function in unpredictable environments. However, alternative explanations should also be considered. In particular, post-pandemic organizational restructuring in healthcare systems, increased workload demands, and rapid digitalization processes may independently contribute to employees’ uncertainty perceptions, beyond leadership effects alone.

When considered alongside previous national and international studies [13, 29, 30], the findings support the general view that leadership and cognitive factors jointly influence adaptation to VUCA conditions. However, this study contributes by integrating these constructs within a healthcare-specific context, where environmental uncertainty, workload pressure, and organizational change frequently coexist.

Overall, the findings suggest that digital leadership should be understood not as a standalone causal driver, but as an enabling organizational factor that interacts with contextual conditions (such as workload, organizational culture, and post-pandemic transformation) and individual cognitive capacities in shaping adaptation to VUCA environments. An important methodological consideration is that the regression analyses were conducted without adjustment for demographic or professional characteristics such as age, occupation, work unit, professional experience, or prior awareness of VUCA. Therefore, the observed associations may partly reflect the influence of unmeasured individual and organizational factors. Future studies should employ adjusted multivariable models to better evaluate the independent contribution of digital leadership to VUCA-related psychological and cognitive dimensions.

## Conclusions

The findings of this study indicate that digital leadership is significantly associated with healthcare professionals’ responses to VUCA-related psychological and cognitive dimensions, particularly resistance to change, uncertainty intolerance, ambiguity tolerance, and cognitive flexibility. The results suggest that higher perceptions of digital leadership are linked with lower resistance to change and uncertainty intolerance, while being positively associated with cognitive flexibility and ambiguity tolerance. These relationships highlight the role of digital leadership as a contextual factor that may support adaptive behavioral and cognitive responses in complex healthcare environments.

From a broader scientific perspective, these findings contribute to the growing body of literature suggesting that leadership in digitally transforming organizations is associated not only with operational and technological outcomes but also with employees’ psychological and cognitive adaptation processes. In healthcare settings, where uncertainty, complexity, and continuous change are inherent characteristics of daily practice, digital leadership may represent an important organizational resource that supports employees’ capacity to navigate challenging work environments.

However, these findings should be interpreted within the scope of the study design, which does not allow for causal inference. Therefore, rather than implying direct causality, the results point to meaningful associations that may reflect the interaction between leadership perceptions and individual adaptive capacities in VUCA conditions.

The relatively stronger associations observed between digital leadership and resistance to change and uncertainty intolerance may indicate that leadership behaviors are particularly relevant in shaping employees’ behavioral and psychological responses to organizational transformation. This finding is especially important for healthcare organizations undergoing rapid digitalization, where employee acceptance of change and the ability to cope with uncertainty are critical for the successful implementation of new technologies and work processes.

Overall, the study provides empirical evidence that digital leadership is associated with several psychological and cognitive responses to VUCA environments among healthcare professionals. While the findings do not establish causal relationships, they support the view that leadership practices, together with individual cognitive resources and organizational conditions, may play a meaningful role in facilitating adaptation within increasingly complex healthcare systems.

Future research should build on these findings by employing longitudinal or experimental designs to better examine causal relationships between digital leadership and VUCA-related psychological and cognitive outcomes. In addition, studies incorporating organizational-level variables such as workload intensity, organizational culture, and digital maturity may provide a more comprehensive understanding of the mechanisms underlying these relationships. Expanding research to different healthcare systems and larger, more diverse samples would also enhance the generalizability of the findings.

### Theoretical implications

This study makes a unique contribution to the health management literature by examining the associations between digital leadership and multiple VUCA-related psychological and cognitive dimensions within a healthcare context. Unlike previous studies that primarily focused on the relationship between digital leadership and organizational performance, innovation, or technology adoption, this study extends the literature by exploring how digital leadership is associated with healthcare professionals’ psychological and cognitive responses to uncertainty and change.

First, the findings provide empirical support for the argument that digital leadership is associated not only with technological transformation processes but also with employees’ adaptive capacities in complex organizational environments. The observed negative associations between digital leadership and both resistance to change and intolerance of uncertainty suggest that digital leadership may be linked to employees’ willingness to engage in organizational change and their ability to function under uncertain conditions. These findings extend current digital leadership literature by highlighting its relevance to individual-level adaptation processes in healthcare organizations.

Second, the positive associations between digital leadership, cognitive flexibility, and ambiguity tolerance contribute to organizational adaptation and resilience theories. Healthcare organizations frequently operate in environments characterized by rapidly evolving technologies, changing patient needs, and increasing uncertainty. The findings suggest that digital leadership may be associated with cognitive and behavioral characteristics that facilitate adaptation in such environments, thereby providing a more comprehensive understanding of how leadership may support workforce resilience in VUCA conditions.

Third, this study contributes to the emerging VUCA literature by providing empirical evidence from healthcare professionals. This population has received relatively limited attention in studies examining digital leadership and VUCA-related psychological and cognitive outcomes. By operationalizing VUCA through resistance to change, intolerance of uncertainty, cognitive flexibility, and tolerance for ambiguity, the study offers a more nuanced understanding of how employees perceive and respond to complexity and uncertainty in healthcare settings.

Overall, the findings strengthen the conceptual linkage between digital leadership, organizational adaptation, and VUCA-related psychological and cognitive constructs, while providing a foundation for future studies investigating the mechanisms through which leadership may influence adaptive responses in digitally transforming healthcare systems.

### Practical implications

The findings of this study suggest that digital leadership may represent an important organizational resource for supporting healthcare professionals’ adaptation to increasingly complex and uncertain work environments. Given the significant associations observed between digital leadership and lower resistance to change and uncertainty intolerance, healthcare organizations may benefit from strengthening leadership practices that promote communication, transparency, and employee engagement during digital transformation initiatives.

In particular, the relatively strong associations between digital leadership and resistance to change indicate that leadership behaviors may play an important role in fostering employees’ readiness to adopt new technologies, workflows, and digitally enabled healthcare practices. This may be especially relevant for healthcare organizations implementing electronic health systems, artificial intelligence applications, telemedicine services, and other digital innovations.

The positive associations between digital leadership, cognitive flexibility, and ambiguity tolerance further suggest that healthcare managers should create organizational environments that encourage learning, problem-solving, and adaptive thinking. Continuous professional development programs, simulation-based training activities, and interdisciplinary learning opportunities may help strengthen employees’ ability to cope with rapidly changing clinical and organizational demands.

Furthermore, healthcare organizations may consider integrating digital leadership competencies into leadership development programs, managerial evaluation frameworks, and strategic workforce planning initiatives. Such efforts may support not only the successful implementation of digital transformation projects but also the development of a workforce that is better prepared to navigate uncertainty and complexity.

Overall, the findings indicate that investments in digital leadership development may contribute to organizational adaptability and workforce preparedness in healthcare systems facing ongoing technological and environmental change.

### Limitations of the research

Several limitations should be considered when interpreting the findings of this study. First, the study was conducted among healthcare professionals working in three hospitals located in the Marmaris district of Muğla, Türkiye. As a result, the findings may not be fully generalizable to healthcare professionals working in different regions, healthcare systems, or organizational contexts.

Second, all data were collected using self-report instruments. Consequently, the findings may be influenced by social desirability bias, response tendencies, or subjective perceptions of the measured constructs. In addition, because all variables were collected from the same respondents at a single point in time, the possibility of common method bias cannot be completely ruled out.

Third, the cross-sectional design of the study precludes causal inference. Therefore, the observed relationships between digital leadership, resistance to change, intolerance of uncertainty, ambiguity tolerance, and cognitive flexibility should be interpreted as associations rather than causal effects. Longitudinal and experimental studies are needed to better understand the direction and underlying mechanisms of these relationships.

Fourth, the regression analyses were unadjusted and did not control for potentially relevant demographic or professional variables, such as age, occupation, work unit, professional experience, and prior awareness of VUCA.

Fifth, the study focused on a limited set of variables and did not include potentially relevant individual and organizational factors, such as digital competencies, organizational culture, technological infrastructure, leadership climate, and team dynamics. The omission of these variables may have reduced the explanatory power of the model.

Finally, the data were collected during a specific period and may therefore reflect contextual influences present at the time of data collection, including organizational workload, technological changes, or situational pressures experienced by healthcare professionals. Future studies involving more diverse samples, multiple data sources, and longitudinal designs would provide a more comprehensive understanding of the relationships examined in this study.

## Data Availability

Data sets used and/or analyzed during the current study are available from the corresponding author on reasonable request.

## Abbreviations

VUCA: Volatility Uncertainty Complexity Ambiguity

## References

[1] Benitez J, Arenas A, Castillo A, Esteves J. Impact of digital leadership capability on innovation performance: The role of platform digitisation capability. Inf Manag. 2022;59(2):103590. 10.1016/j.im.2022.103590.

[2] Deloitte. Leading in a VUCA world. Deloitte Insights. 2019. https://www2.deloitte.com/us/en/pages/technology-media-and-telecommunications/articles/leading-in-a-vuca-world.html.

[3] Bennett N, Lemoine GJ. What a difference a word makes: Understanding threats to performance in a VUCA world. Bus Horiz. 2014;57(3):311–7. 10.1016/j.bushor.2014.01.001.

[4] Nowacka A, Rzemieniak M. The impact of the VUCA environment on the digital competences of managers in the power industry. Energies. 2021;15(1):185. 10.3390/en15010185.

[5] Dhir S, Sushil, editors. Flexible strategies in VUCA markets. Singapore: Springer; 2018. 10.1007/978-981-10-8926-8.

[6] Horstmeyer A. The generative role of curiosity in soft skills development for contemporary VUCA environments. J Organ Change Manage. 2020;33:737–51. 10.1108/JOCM-08-2019-0250.

[7] Cernega AC, Nicolescu DN, Meleșcanu Imre M, Ripszky Totan A, Arsene AL, Șerban RS, Perpelea A-C, Nedea M-I, Pițuru S-M. Volatility, uncertainty, complexity, and ambiguity (VUCA) in healthcare. Healthcare. 2024;12(7):773. 10.3390/healthcare12070773. 38610195 PMC11011466

[8] Shin J, Mollah M, Choi J. Sustainability and organizational performance in South Korea: the effect of digital leadership on digital culture and employees’ digital capabilities. Sustainability. 2023;15:2027. 10.3390/su15032027.

[9] Laukka E, Pölkki T, Kanste O. Leadership in the context of digital health services: A concept analysis. J Nurs Adm Manag. 2022;30(7):2763–80. 10.1111/jonm.13763. PMC10087820 35942802

[10] Kludacz-Alessandri M, Hawrysz L, Żak K, Zhang W. The impact of digital transformational leadership on digital intensity among primary healthcare entities: A moderated mediation model. BMC Health Serv Res. 2025. 10.1186/s12913-025-12283-x., 25, Article 12283. 39838353 PMC11752656

[11] Weber E, Büttgen M, Bartsch S. How to take employees on the digital transformation journey: An experimental study on complementary leadership behaviors in managing organizational change. J Bus Res. 2022;143:225–38. 10.1016/j.jbusres.2022.01.036.

[12] Gao P, Gao Y. How Does Digital Leadership Foster Employee Innovative Behavior: A Cognitive–Affective Processing System Perspective. Behav Sci. 2024;14(5):362. 10.3390/bs14050362. 38785853 PMC11117572

[13] Guo Y, Zhao Q, Cao Z, Huang S. The influence of tourism and hospitality students’ perceived effectiveness of outcome-based education on their VUCA skills. Sci Rep. 2023;13(1):8079. 10.1038/s41598-023-35055-8. 37202468 PMC10193308

[14] Goodfellow L. An overview of survey research. Respir Care. 2023;68:1309–13. 10.4187/respcare.11041. 37072162 PMC10468179

[15] Claassen K, Anjos D, Kettschau DR, J., Broding HC. How to evaluate digital leadership: A cross-sectional study. J Occup Med Toxicol. 2021;16(1):44. 10.1186/s12995-021-00335-x. 34598724 PMC8485107

[16] Bilginoğlu E, Yozgat U. The validity and reliability of the measure for digital leadership: Turkish form. In: Vardarlıer P, editor. Multidimensional and strategic outlook in digital business transformation. Cham: Springer; 2023. p. 53–67. 10.1007/978-3-031-23432-3_5.

[17] Koç M, Özekenci E, Bir Y. VUCA bileşenlerinin ölçümlenmesi üzerine bir ölçek uyarlama çalışması. In: 20. Uluslararası İşletmecilik Kongresi Bildiriler Kitabı. 2022.

[18] Oreg S. Personality, context, and resistance to organizational change. Eur J Work Organizational Psychol. 2007;15(1):73–101. 10.1080/13594320500451247.

[19] Carleton RN, Norton MPJ, Asmundson GJ. Fearing the unknown: A short version of the Intolerance of Uncertainty Scale. J Anxiety Disord. 2007;21(1):105–17. 10.1016/j.janxdis.2006.03.014. 16647833

[20] Martin MM, Rubin RB. A new measure of cognitive flexibility. Psychol Rep. 1995;76(2):623–6. 10.2466/pr0.1995.76.2.623.

[21] McLain DL. Evidence of the properties of an ambiguity tolerance measure: The multiple stimulus types ambiguity tolerance scale–II (MSTAT–II). Psychol Rep. 2009;105(3):975–88. 10.2466/PR0.105.3.975-988. 20099561

[22] Gürbüz S, Şahin F. *Sosyal bilimlerde araştırma yöntemleri*, vol. 271. Ankara: Seçkin Yayıncılık; 2018; pp. 74–82.

[23] Avolio BJ, Kahai SS, Dodge GE. E-leadership: Re-examining transformations in leadership source and transmission. Leadersh Q. 2014;25(1):105–31. 10.1016/j.leaqua.2013.11.003.

[24] Kahre C, Hoffmann D, Ahlemann F. Beyond business-IT alignment: digital business strategies as a paradigmatic shift. Proc 50th Hawaii Int Conf Syst Sci. 2017:4706–4715. 10.24251/HICSS.2017.568.

[25] Kotter JP. Leading change. Boston (MA): Harvard Business Review Press; 2012.

[26] Ye Q. Digital leadership enhances organizational resilience by fostering job crafting: The moderating role of organizational culture. Sci Rep. 2025;15:Article 09144. 10.1038/s41598-025-09144-2. PMC12241576 40634440

[27] Saif N, Ali S, Shaheen I, Goh G, Khan S. Revolutionising healthcare leadership: the critical role of digital citizenship in knowledge sharing. Sci Rep. 2025;15:93117. 10.1038/s41598-025-93117-y. PMC11910507 40089554

[28] Malik M, Raziq M, Sarwar N, Tariq A. Digital leadership, business model innovation, and organisational change: Role of the leader in steering digital transformation. Benchmarking: Int J. 2024. 10.1108/BIJ-04-2023-0283.

[29] Çiçek KS. VUCA dünyasında kuantum liderlik (Yayımlanmamış yüksek lisans tezi). İstanbul Gedik Üniversitesi Lisansüstü Eğitim Enstitüsü, İşletme Yönetimi Anabilim Dalı. Türkiye: İstanbul; 2023.

[30] Tarsuslu S. Sağlık çalışanlarının COVID-19 sürecinde karşılaştıkları VUCA ortamının iş stresi ve tükenmişlikleri üzerindeki etkisi. Anadolu Univ J Econ Administrative Sci. 2023;24(3):399–419. 10.53443/anadoluibfd.1261059.

